# Depressive symptoms and daily living dependence in older adults with type 2 diabetes mellitus: the mediating role of positive and negative perceived stress

**DOI:** 10.1186/s12888-023-05273-y

**Published:** 2024-01-02

**Authors:** Li Ai Tai, Le Yu Tsai, Chia Hung Lin, Yi Chen Chiu

**Affiliations:** 1 Graduate Institute of Clinical Medical Science, College of Medicine, Chang Gung University, Taoyuan, Taiwan; 2 Department of Nursing, Cardinal Tien Junior College of Healthcare and Management, New Taipei, Taiwan; 3 Department of Endocrinology and Metabolism, Yonghe Cardinal Tien Hospital, New Taipei, Taiwan; 4 Division of Endocrinology and Metabolism, Department of Internal Medicine, Chang Gung Memorial Hospital, Linkou, Taoyuan, Taiwan; 5 College of Medicine, Chang Gung University, Taoyuan, Taiwan; 6 School of Nursing, College of Medicine, Chang Gung University, Taoyuan, Taiwan

**Keywords:** Type 2 Diabetes Mellitus, Depressive symptoms, Perceived stress, Instrumental activities of daily living, Mediation

## Abstract

**Background:**

Higher stress is associated with higher levels of depression and instrumental-activities-of-daily-living (IADL) dependence, and depression is strongly associated with specific IADL disabilities. Accordingly, the aim of this study was to investigate the mediating effect of perceived stress on the association between depression and IADL dependence among older adults with diabetes mellitus (DM).

**Methods:**

We examined baseline data collected from a longitudinal study that recruited 110 patients with DM aged ≥ 65 years from the endocrinology outpatient clinic of a district hospital. The instruments used for our measurement processes comprised a demographic data sheet and Chinese versions of the Perceived Stress Scale (PSS), the short form of the Geriatric Depression Scale (GDS-S), and the Lawton IADL Scale. We assessed the mediating effects of positive perceived stress (PPS) and negative perceived stress (NPS) after controlling for five covariates by using a regression-based model run through the SPSS macro PROCESS.

**Results:**

We observed negative correlations between GDS-S scores and PPS and between PPS and IADL dependence; we noted positive correlations between GDS-S scores and NPS and between NPS and IADL dependence (all *P* < 0.01). The indirect effect is coefficient = 0.12, [95% confidence interval = (0.0, 0.33)], suggesting that PPS achieves a mediating effect between depressive symptoms and IADL dependence. However, the NPS does not achieve a mediating effect in the relationship between depressive symptoms and IADL dependence (coefficient = 0.06, 95% CI = − 0.03, 0.15).

**Conclusions:**

Personal PPS mediates the association between depression and IADL dependence in older adults with DM. This finding suggests that providing patients with psychological education to promote their PPS may help prevent their functional decline.

## Background

The number of people with diabetes mellitus (DM) is expected to increase to 640 million by 2040. DM is a metabolic disease characterized by an abnormally elevated blood glucose level [1, 2], and among individuals with DM, 50–90% are at an increased risk of several types of disabilities and 23% are dependent regarding instrumental activities of daily living (IADLs). In particular, older patients with DM (aged ≥ 65 years) were reported to exhibit a higher prevalence of mobility and IADL dependence relative to other populations with DM [3]; moreover, more than 40% of such patients reported difficulties in performing heavy housework [4]. Disability is associated with increased mortality [5, 6] and depression [7] and a decreased quality of life [7, 8]. Therefore, disability places a heavy burden on the global health-care system [5]. Disability typically begins with an increase in IADL dependence [9, 10]. In addition, a study reported a direct correlation between depressive symptoms and perceived stress, which may lead to greater IADL dependence [11].

Depression is an emotional and psychological state characterized by feelings of sadness and hopelessness and a consistently low mood; hopelessness and sadness are key factors that hinder independent living in older adults [8]. Approximately 80% of individuals with DM experience depression during disease progression [12]. Those with depression may exhibit poor disease control, medication use, and social interaction, and may experience difficulties with personal mobility or the performance of independent self-care tasks [13–16]. Depression resulting from long-term blood sugar control and treatment [9, 10], affects a patient’s dietary and medication behaviors [11, 12].

Perceived stress is defined as an individual’s subjective perception of internal and external stress events [17]. Empirical evidence indicates that stress due to chronic illness affects an individual’s participation in daily activities [18]. Chronic psychological stress can accelerate biological aging; specifically, high stress responses are often accompanied by depression that directly or indirectly affects a patient’s adaptation to or management of their disease and their ability to cope with stress [19]. Studies have discovered that chronic stress in individuals with DM can affect their disease prognosis and self-care behaviors, including their dietary control, blood sugar control, physical activity, and adherence to medication and lifestyle modifications [20]. Therefore, perceived stress in individuals with DM is significantly correlated with IADL dependence [8].

A study suggested that declines in physical and mental health increase perceptions of stress among older patients [21]. Perceived stress comprises positive and negative dimensions [22]. Different types of perceived stress may be independently perceived as threatening or requiring adaptive adjustment [17, 19, 22–24]. These different stress appraisals lead to the development of different behavioral coping strategies [25, 26] which can influence an individual’s response to a life event. For example, negative perceived stress (NPS) can induce an individual to adopt an emotion-focused coping strategy that involves maladaptive coping mechanisms. Therefore, NPS can affect an individual’s IADL dependence [27, 28]. By contrast, positive perceived stress (PPS) involve a positive appraisal of stressful situations and can prompt an individual to adopt a problem-solving strategy that influences their self-care behaviors. Evidence demonstrates that stressful situations may affect the blood sugar control and physical activity levels of individuals with DM and that perceived stress patterns may affect their stress-coping strategies and self-care. Additionally, the mentioned study confirmed the association between depressive symptoms and physical health and validated the mediating role of perceived stress in this association [21]. Overall, perceived stress is regarded as a mediator in the association between mental and physical health [28, 29]. However, there are very few studies specifically to address the relationships between depression, perceived stress (PPS and NPS) and IADL dependence among older adults with DM. Accordingly, to fill this research gap, the present study comprehensively investigated whether positive and negative perceptions of stress mediate the association between depressive symptoms and IADL dependence in the target population. Our findings can inform future research on the development of personalized interventions aiming at managing perceived stress among older adults with DM.

## Methods

### Study population and procedures

This study employed a cross-sectional design to examine data derived from the baseline data of a longitudinal study. In that longitudinal study, research data were collected between June 2017 and August 2018, and the study was conducted at the outpatient endocrinology clinic of a hospital in northern Taiwan (Project Number: R106-003) [30]. The longitudinal study was approved by an institutional review board (IRB; IRB number, CTB-106-3-5-011) and was conducted in accordance with the Declaration of Helsinki to protect the rights and well-being of its participants. The purpose of the longitudinal study was to explore multiple risk factors for IADL dependence in older adults with DM and to examine predictors of changes in IADL independence. All candidates were assessed by an attending physician, and eligible candidates were then selected as participants by the investigators. Before completing a questionnaire survey, all participants were informed of the purpose of the study, asked to provide informed consent, and assured that their participation in the study was completely voluntary, anonymous, and confidential.

The inclusion criteria were as follows: (1) being aged ≥ 65 years, (2) having a diagnosis of type 2 DM, and (3) being able to communicate in Mandarin, Taiwanese, or Hakka. The exclusion criteria were as follows:(1) having a terminal illness or (2) scoring < 20 points on the Mini-Mental State Examination [31]. After data collection was completed, the collected data were exported to Microsoft Excel and subsequently imported into IBM SPSS Statistics for Windows (Version 21.0; IBM, Armonk, NY, USA) for data management and analysis.

### Measures

#### Questionnaire for collecting demographic information

A questionnaire was applied to collect information from the participants. The questionnaire required approximately 30–40 min to complete, and it included a demographic information sheet, the Geriatric Depression Scale-Short Form (GDS-S), the Perceived Stress Scale (PSS), and a modified version of the original Lawton IADL Scale [32]. The collected demographic data comprised information on the participants’ sex, age, educational level, and marital status; the number of chronic diseases that a participant had; whether a participant implemented diabetic dietary control (“yes” or “no” response); and whether a participant exercised regularly (“yes” or “no” response). Considering that caregivers can affect the IADL dependence of patients with DM, binary options (“yes” or “no” response) were provided to enable the participants to clarify their status with respect to daily activity dependence, including whether their primary caregiver was their spouse or child, it relied on patients to provide information about their family caregivers. Furthermore, the present study collected patients’ clinical data on the status of participants’ DM-related neuropathy examined and determined by their physicians. We also collected the total number of diseases that the participants had by considering their systemic disease diagnoses as indicated in their electronic medical records.

The GDS-S comprises 15 questions for evaluating depression in older people, and each question is answered with a “yes” (scored as 1) or “no” (scored as 0) response. The overall score for the GDS-S ranges from 0 to 15, and a higher total score indicates more severe depressive symptoms. A previous study reported that a Chinese version of the GDS-S could correctly distinguish pproximately > 90% of depression cases from noncases in a community survey [33]. Specifically, the Chinese version of the GDS-S was reported to have internal consistency reliability (Cronbach’s *α*) values between 0.89 [34] to 0.94 [35] and a test–retest reliability (*r*) of 0.85 [35]. Therefore, the present study used this Chinese version of the GDS-S because of its sound psychometric properties. In the present study, the Cronbach’s *α* value of the scale was 0.80.

The 14-item Chinese version of the PSS is a self-rated scale that measures the stress experienced by an individual in the preceding month on a 5-point Likert scale (0, *never*; 1, *occasional*; 2, *sometimes*; 3, *often*; 4, *always*). Seven of the 14 PSS items are worded negatively (items 1, 2, 3, 8, 11, 12, and 14) to form an NPS subscale; the remaining seven are worded positively (items 4, 5, 6, 7, 9, 10, and 13) to form a PPS subscale. The overall score for the PSS is calculated after reversing the scores for the positive items and then summing the scores for all items to obtain an overall score ranging between 0 and 56 [25]; a higher score indicates a greater stress. The scale was reported to have a Cronbach’s *α* value of 0.84–0.86 and a test–retest reliability of 0.85 [9, 25]. The Cronbach’s *α* values of the PSS scale, PPS subscale, and NPS subscale used in the present study were 0.76, 0.80, and 0.71, respectively.

A modified version of the original Lawton IADL Scale [32] was used to evaluate the IADL dependence of the participants; the modified scale comprises eight items, which pertain to shopping, using the telephone, housekeeping activities, doing laundry, preparing food, using transportation, handling medications, and handling finances. The original scale comprises nine items, whereas the modified version comprises eight items. Specifically, questions 4 (pertaining to doing heavy work at home or nearby) and 5 (pertaining to sweeping, washing dishes, taking out the trash, and other light chores) were combined into a single question, which refers to the activities mentioned in questions 4 and 5 collectively as “housework.” We modified the scoring system such that each item is rated between 0 and 3 (0, *independent*; 1, *some dependence*; 2, *very dependent*; 3, *complete dependence*), for a total score ranging between 0 and 24. In the present study, the scale was determined to have a reliability of 0.8–0.9 [32], a test–retest reliability of 0.90, an internal consistency of 0.86 [36], and a Cronbach’s *α* value of 0.90.

### Statistical analysis

**Fig. 1:**
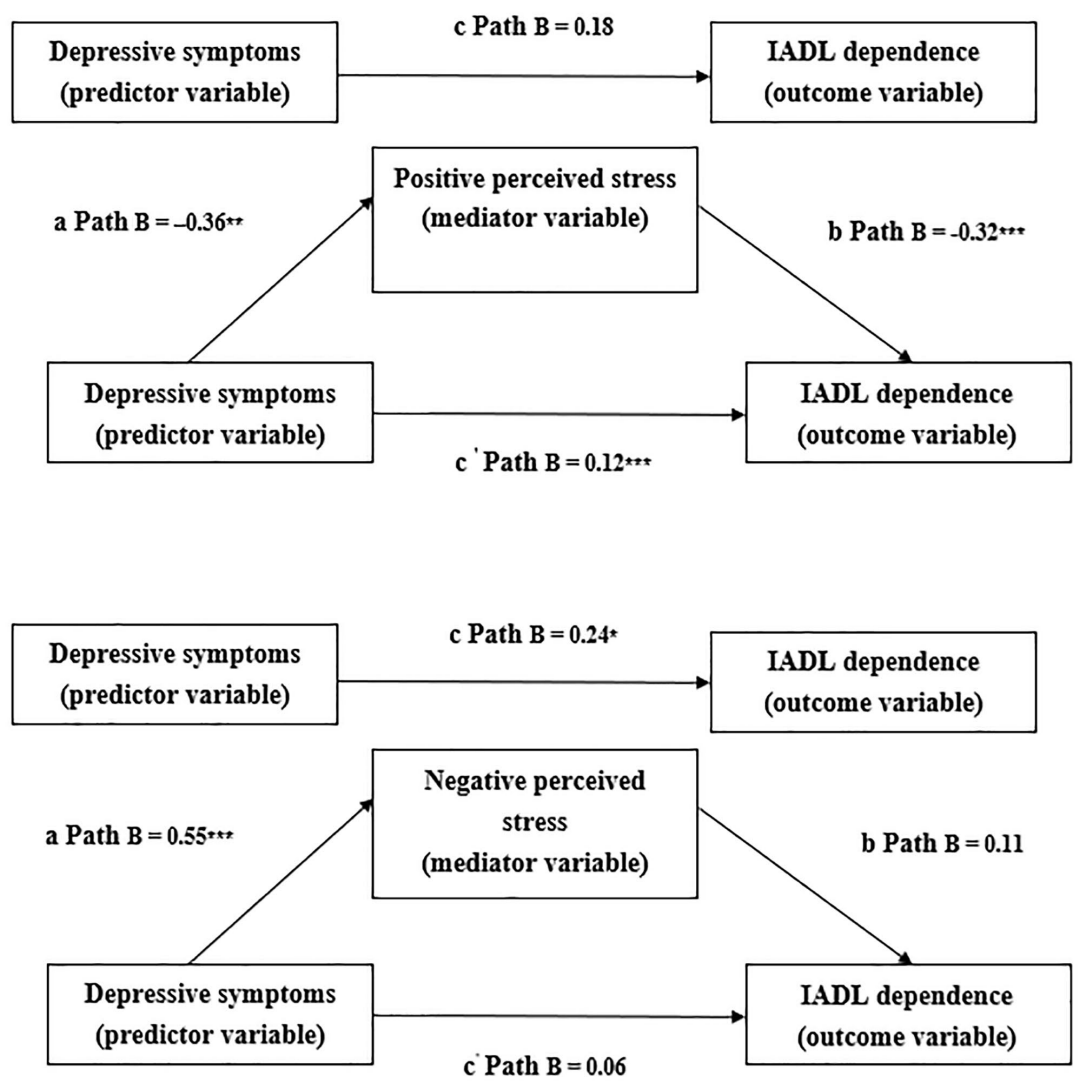
Mediating effects of positive and negative perceived stress ^*^P < 0.05, ^**^P < 0.01, ^***^P < 0.001

Statistical analyses were conducted using SPSS version 21.0. Descriptive, categorical variables were expressed as frequency and percentage values, and continuous variables were expressed as mean and standard deviation (SD) values. The analysis strategies are described as follows: (1) Pearson correlation was used to determine relationships among variables. (2) A multiple regression model was used to determine predictive relationships among depressive symptoms, IADL dependence, and perceived stress after adjustment for several demographic variables (i.e., age, exercise, dietary control for DM, neuropathy, and total number of diseases). (3) Through the PROCESS macro in SPSS, we used a multiple regression model to verify the mediating effects of perceived stress on the association between depressive symptoms and IADL dependence [37]. The two-step method developed by Baron and Kenny [38] was applied to evaluate the direct and indirect effects of perceived stress on IADL dependence. First, a multiple regression model of IADL dependence was used to preliminarily measure the mediating effects of perceived stress. Second, the bootstrapping method, as defined by Preacher and Hayes [39], was adopted to test the indirect effects of resilience on IADL dependence. Notably, the results from these analyses revealed significant correlations among the three variables: depressive symptoms, perceived stress (PPS and NPS), as well as IADL independence. The bootstrapping process involved 5,000 repetitions, and 95% confidence intervals (CIs) were calculated. If the range for a 95% CI did not encompass zero, an indirect link was regarded as significant [40]. In the present study, we regarded a *P* value of < 0.05 (two-tailed) as a significant result. Baron and Kenny [38] presented the conditions for mediation as a pathway diagram (Fig. 1).

To determine the role of perceived stress state as a mediator, we employed two methods. First, we conducted a multiple regression analysis in which demographic variables served as covariates. Second, we used the PROCESS macro [40] and employed one independent variable (depressive symptoms), two mediators (PPS and NPS), and one dependent variable (IADL dependence). The regression coefficients *a* (depressive symptoms), *b* (PPS and NPS), and *c* (depressive symptoms–IADL dependence) were revealed to be statistically significant, and pathway *c*’ (depressive symptoms–IADL dependence while controlling for PPS and NPS) was calculated. Finally, the mediating effects of PPS and NPS on the association between depressive symptoms and IADL dependence was tested (pathways *c* and *c’*). Subsequently, depressive symptoms were used as the predictive variable for predicting IADL dependence (pathway *c* in Fig. 1).

## Results

### Characteristics of participants and distribution of depressive symptoms, perceived stress, and IADL dependence

Of the 110 participants (Table 1), 68 were women (61.8%). The mean age of the participants was 73.43 years (SD = 6.91 years). Of the participants, 52 (47.3%) exercised regularly, 57 (51.8%) adhered to a diabetic dietary plan, 40 (36.4%) had neuropathy, and 74 (67.3%) had their spouses as their primary caregivers. Furthermore, the mean number of comorbidities among the participants was 2.8 (SD = 1.14), and the mean GDS-S, PPS, NPS, and IADL dependence scores were 3.94 (SD = 3.27), 18.34 (SD = 4.68), 10.48 (SD = 4.12), and 2.53 (SD = 4.46), respectively. The participants examined in the present study were all home based.

**Table 1:** Characteristics of participants and distribution of depressive symptoms, perceived stress, and IADL dependence (N = 110)

Variables	N(%)	Mean ± SD
Personal characteristic factors		
Age		73.43 ± 6.91
Female gender	68 (61.8)	
Married	92 (83.6)	
Years of education		9.06 ± 4.18
Regular exercise (Yes)	52 (47.3)	
Diabetic dietary control (Yes)	57 (51.8)	
Source of primary caregivers reported by patients		
Spouses (Yes)	74 (67.3)	
Children (Yes)	36 (32.7)	
Neuropathy (Yes)	40 (36.4)	
Disease (n.)		2.75 ± 1.14
>2 diagnoses	48 (43.6)	
GDS-S (0–15 points)		3.94 ± 3.27
Perceived Stress Scale		
PPS (0–28 points)		18.34 ± 4.68
NPS (0–28 points)		10.48 ± 4.12
Outcome variable		
IADL (0–24 points) dependence		2.53 ± 4.46

Note: Disease (n.): total number of diseases; GDS-S: Geriatric Depression Scale-Short Form (0–15 points); PPS: positive perceived stress (0–28 points); NPS: negative perceived stress (0–28 points); IADLs: instrumental activities of daily living (0–24 points)

### Correlations between depressive symptoms, perceived stress, and IADL dependence

Results showed that a positive correlation between the GDS-S and IADL dependence (*r* = 0.39, *P* < 0.01), a negative correlation between the GDS-S and PPS (*r* = − 0.36, *P* < 0.01), a positive correlation between the GDS-S and NPS (*r* = 0.49, *P* < 0.01), a negative correlation between PPS and IADL dependence (*r* = − 0.56, *P* < 0.01), and a positive correlation between NPS and IADL dependence (*r* = 0.34, *P* < 0.01).

### Mediating effects of perceived stress on association between depressive symptoms and IADL dependence

We employed a multiple regression model and bootstrapping sampling to test the indirect effect of depressive symptoms on IADL dependence through perceived stress. Table 2 lists the results obtained from the multiple regression model. Specifically, Model 1 revealed that depressive symptoms were significantly associated with IADL dependence, with the standardized regression coefficient β being 0.223 (*P* = 0.007). In Model 2, we included PPS in the mediating model and found a significant association between depressive symptoms and IADL dependence (β= −0.338, *P* < 0.001); when PPS was added, the absolute value of β for depressive symptoms decreased significantly from 0.223 to 0.138, preliminarily demonstrating the mediating role of PPS (Table 2).

**Table 2:** Results from multiple regression model of IADL dependence (N = 110)

Variables	Model 1			Model 2		
PPS	*β* (SE)	*t*	*P*	*β* (SE)	*t*	*P*
Constant	-18.424(3.752)	-4.910	< 0.001	-6.860(4.491)	-1.527	0.130
GDS-S	0.223(0.110)	2.777	0.007	0.138(0.106)	1.771	0.080
PPS				-0.338(0.078)	-4.099	< 0.001
	*R*^2^ = 0.410, *F =* 7.713, *P* = 0.007			*R*^2^ = 0.493, *F =* 14.182, *P* = < 0.001		
				Adjustment *R*^*2*^ = 0.458		
				*R*^*2*^ change = 0.083		
				* F* change = 16.799^***^		
NPS	Model 1			Model 2		
	*β* (SE)	*t*	*P*	*β* (SE)	*t*	*P*
Constant	-18.424(3.752)	-4.910	< 0.001	-18.661(3.757)	-4.967	< 0.001
GDS-S	0.223(0.110)	2.777	0.007	0.182(0.122)	2.027	0.045
NPS				0.093(0.097)	1.048	0.297
	*R*^2^ = 0.410, *F =* 7.713, *P* = 0.007			*R*^2^ = 0.416, *F =* 10.382, *P* = < 0.001		
				Adjustment *R*^*2*^ = 0.376		
				*R*^*2*^ change = 0.006		
				* F* change = 1.097		

Note: *β* = standardized regression coefficient; SE = standard error. Control variables: age, exercise, diabetic dietary control, and neuropathy. Disease (n.): total number of diseases; GDS-S: Geriatric Depression Scale-Short Form (0–15 points); PPS: positive perceived stress (0–28 points); NPS: negative perceived stress (0–28 points); IADLs: instrumental activities of daily living (0–24 points)

Regarding NPS, Model 1 revealed that depressive symptoms were significantly associated with IADL dependence, with the standardized regression coefficient β being 0.223, (*P* = 0.007). In Model 2, we included NPS in the mediation model and observed a significant association between depressive symptoms and IADL dependence (β = 0.182, *P* = 0.045). When NPS was added, the absolute value of β decreased from 0.223 to 0.182, but the mediating effect of NPS was nonsignificant (*P* = 0.297).

Through the PROCESS macro (Table 3; Fig. 1), we observed both the direct (coefficient = 0.18, 95% CI = − 0.03, 0.39), and indirect (coefficient = 0.12, 95% CI = 0.0, 0.33]) effects of PPS, suggesting that PPS had a mediating effect on the association between depressive symptoms and IADL dependence.

**Table 3:** Analysis of direct and indirect effects (bootstrap estimation)

Variables					
**Pathway**	Coefficient	SE	*P*	Bootstrap 95% CI	
**PPS**				Lower	Upper
**Direct effects**					
GDS-S—IADLs	0.18	0.11	0.08	-0.03	0.39
**Indirect effects**					
GDS-S-—PPS —IADLs	0.12	0.08	< 0.001	0.01	0.33
**Pathway**	Coefficient	SE	*P*	Bootstrap 95% CI	
**NPS**				Lower	Upper
**Direct effects**					
GDS-S—IADLs	0.24	0.12	0.05	0.00	0.48
**Indirect effects**					
GDS-S-—NPS —IADLs	0.06	0.05	0.02	-0.03	0.15

Note: B = unstandardized regression coefficient; SE = standard error; CI = confidence interval

Through PROCESS, we also determined the direct (coefficient = 0.24, 95% CI = 0.00, 0.48) and indirect (coefficient = 0.06, 95% CI = − 0.03, 0.15) effects of NPS. However, the mediating effect of NPS on the association between depressive symptoms and IADL dependence was nonsignificant because it was not within the 95% CI (Table 3; Fig. 1).

## Discussion

According to our literature review, the present study is the first to clarify the mediating effects of PPS on the relationship between depressive symptoms and IADL dependence in older adults with DM. The findings of the present study reveal negative correlations between the GDS-S scores and PPS and between PPS and IADL dependence; they also indicate positive correlations between the GDS-S scores and NPS and between NPS and IADL dependence. Collectively, these findings indicate that different types of perceived stress played different mediating roles and that PPS fully mediated the association between depressive symptoms and IADL dependence. However, the mediating effect of NPS on the association between depressive symptoms and IADL dependence was nonsignificant.

Therefore, our results regarding the significant mediating effect of PPS demonstrate the future development of positive psychological interventions for such patients.

### Correlations between depressive symptoms, perceived stress, and IADL dependence

Depressive symptoms are associated with exposure to stressful conditions, including psychological distress, emotional disorders, and negative personality traits such as anger and hostility [41]. Empirical evidence strongly supports the association between depression and disability in the general older adult population [42]. In addition, a study involving older adults with DM revealed that those with depressive symptoms exhibited more severe disabilities than did those without such symptoms [43]. Therefore, the results of the present study suggest that clinical interventions aiming at reducing depressive symptoms should be implemented to help promote IADL independence.

### Mediating role of perceived stress state on depressive symptoms and IADL dependence

According to our results, the severity of PPS mediated the relationship between depressive symptoms and IADL dependence, whereas NPS directly influenced the relationship between depressive symptoms and IADL dependence. We inferred that an improvement in PPS can reduce IADL disability by reducing obstacles related to depressive symptoms. Moreover, our study validated the findings of a meta-analysis that reported that the presence of comorbid depression in individuals with chronic diseases affects their medication self-management and physical activity behaviors [44, 45] and that IADL dependence is significantly associated with depression [46]. Nonpharmaceutical interventions that can reduce emotional stress, such as interventions involving physical activity [47], are also key moderators for improving the physical health of patients [48]. Furthermore, our study extends the literature by using the PPS as a mediator to impact the relationship between depressive symptoms and IADL dependence [41, 44–46] because of the effects of psychological pressure; by contrast, NPS had no such effect.

Among the examined variables, perceived stress was identified as a key factor influencing IADL dependence. Individuals with higher levels of perceived stress have been reported to be more prone to negative reappraisals [49]. Psychosocial and environmental interventions can help prevent or alleviate negative emotions [50]. Therefore, on the basis of the results of the present study, we propose that problem-oriented stress management strategies should be taught to patients who are undergoing treatment for DM and exhibiting depressive symptoms. These strategies can improve their perceived stress, prevent or alleviate their negative emotions, and prevent them from developing IADL dependence.

Studies have indicated that perceived stress is linked to physical inactivity [51], that patients with physical impairments and/or disabilities exhibit high levels of perceived stress [21], and that social support can help individuals to cope with stress, with psychological support playing a crucial role in this process [52]. In addition, personality and attitudes can contribute to an individual’s ability to cope with stress, which in turn influences the severity of their depressive symptoms; in this context, a positive attitude can be cultivated through the utilization of personal and environmental resources [53, 54]. One study reported that when patients experienced depressive symptoms due to long-term exposure to external stressors, they tended to struggle with self-care and exhibit decreased activity levels and poor dietary behaviors or to experience difficulties with medication use [54]. On the basis of our findings (Fig. 1), we suggest that clinicians routinely assess the psychological health of patients with DM and provide individual counseling services for those who experience emotional distress; doing so can prevent the stress and disability engendered by DM as a long-term chronic illness.

A possible explanation for the nonsignificant effects of NPS in the present study is that the cross-sectional design which might be insufficient for observing the changes in its effect on negative emotions. Another explanation is that the direct influence of depression on IADL dependence outweighed the influence of NPS on IADL dependence (Tables 4 and 2). Both self-perceived stress and depressive emotions—representing the subjective perceptions of individuals—are likely to be influenced by age and cognitive factors. Therefore, more research should be conducted to re-examine the mediating effect of NPS on the relationship between depressive symptoms and IADL dependence in older patients with DM.

**Table 4:** Correlations between depressive symptoms, perceived stress, and IADL dependence (N = 110)

Variables	Age	Education level	Diseases (n.)	GDS-S	PPS	NPS	IADLs
Age	1						
Education level	-0.12	1					
Diseases (n.)	0.05	-0.09	1				
GDS-S	0.22^*^	-0.01	-0.01	1			
PPS	-0.38^**^	0.05	-0.03	-0.36^**^	1		
NPS	0.24^*^	-0.03	-0.07	0.49^**^	-0.23^*^	1	
IADLs	0.51^**^	-0.14	-0.01	0.39^**^	-0.56^**^	0.34^**^	1

^*^P < 0.05, ^**^P < 0.01

### Limitations

The present study had several limitations. First, the present study analyzed only outpatient cases and did not include hospitalized patients who tend to experience greater levels of stress and dependence in relation to activities of daily living. Second, the sample of the present study comprised only patients from a single community hospital who were enrolled through convenience sampling. Therefore, the generalizability of the present study’s results is likely limited. Third, because of the use of self-reported measures, the results were unavoidably affected by participant response bias. Although the effect of NPS was nonsignificant, we must still consider the characteristics and cognitive function of each patient as well as the effects of subjective stress perceptions and emotional distress on IADLs. Therefore, we recommend routinely clinical monitoring of cognitive function, depressive symptoms, NPS and IADL dependency in these patients.

## Conclusion

The key finding of the present study is that PPS fully mediates the association between depressive symptoms and IADL dependence in older adults with DM. An improvement in positive emotions can reduce the effects of depressive symptoms on IADL dependence; thus, the effects of perceived stress on depressive symptoms in this population should not be ignored. In future studies, we intend to explore the mediating effect of perceived stress state, which is a key research area.

### Relevance for clinical practice

The present study clarified the mediating effects of various types of perceived stress on the association between depression and IADL dependence. In clinical care, strengthening interventions aimed at reducing disease-related stress in patients and facilitating patients’ adaptation to their conditions can help alleviate their negative emotions. PPS plays a mediating role in improving activities of daily living, whereas NPS may interfere with depressive symptoms and aggravate DM-related dysfunction. Therefore, psychological counseling is required in clinical care to alleviate chronic disease–related stress and negative emotions. Individualized measures should be introduced to encourage the adoption of a problem-focused positive attitude among patients with chronic diseases, particularly older adults with DM, thereby helping them alleviate their depression symptoms and reduce their IADL dependence.

## Data Availability

The datasets used and/or analyzed during the current study are available from the corresponding author on reasonable request.

## References

[1] Klein KR, Buse JB (2020). The trials and tribulations of determining HbA1c targets for Diabetes Mellitus. Nat Rev Endocrinol.

[2] Kowluru RA, Kowluru A, Mishra M, Kumar B (2015). Oxidative stress and epigenetic modifications in the pathogenesis of diabetic retinopathy. Prog Retin Eye Res.

[3] Cowie CC, Casagrande SS, Menke A, Cissell MA, Eberhardt MS, Meigs JB et al. Diabetes Am. 2018.

[4] Martin LG, Zimmer Z, Hurng BS (2011). Trends in late-life disability in Taiwan, 1989–2007: the roles of education, environment, and technology. Popul Stud.

[5] James SL, Abate D, Abate KH, Abay SM, Abbafati C, Abbasi N (2018). Global, regional, and national incidence, prevalence, and years lived with disability for 354 Diseases and injuries for 195 countries and territories, 1990–2017: a systematic analysis for the global burden of Disease Study 2017. Lancet.

[6] Barbour KE, Lui LY, McCulloch CE, Ensrud KE, Cawthon PM, Yaffe K (2016). Trajectories of lower extremity physical performance: effects on fractures and mortality in older women. J Gerontol Series A Biomed Sci Med Sci.

[7] Lamb VL (1996). A cross-national study of quality of life factors associated with patterns of elderly disablement. Soc Sci Med.

[8] Tsai YH, Chuang LL, Lee YJ, Chiu CJ (2021). How does Diabetes accelerate normal aging? An examination of ADL, IADL, and mobility disability in middle-aged and older adults with and without Diabetes. Diabetes Res Clin Pract.

[9] Yang T, Huang H (2003). An epidemiological study on stress among urban residents in social transition period. Zhonghua Liu Xing Bing Xue Za Zhi = Zhonghua Liuxingbingxue Zazhi.

[10] Hung WW, Ross JS, Boockvar KS, Siu AL (2011). Recent trends in chronic Disease, impairment and disability among older adults in the United States. BMC Geriatr.

[11] Shahimi NH, Goh CH, Mat S, Lim R, Koh VCA, Nyman SR (2022). Psychological status and physical performance are independently associated with autonomic function. Biomed Eng Online.

[12] Katon W, Von Korff M, Ciechanowski P, Russo J, Lin E, Simon G (2004). Behavioral and clinical factors associated with depression among individuals with Diabetes. Diabetes Care.

[13] Restivo MR, McKinnon MC, Frey BN, Hall GB, Syed W, Taylor VH (2017). The impact of obesity on neuropsychological functioning in adults with and without major depressive disorder. PLoS ONE.

[14] Wong E, Backholer K, Gearon E, Harding J, Freak-Poli R, Stevenson C (2013). Diabetes and risk of physical disability in adults: a systematic review and meta-analysis. Lancet Diabetes Endocrinol.

[15] Paterson B, Thorne S, Crawford J, Tarko M (1999). Living with Diabetes as a transformational experience. Qual Health Res.

[16] Abdi S, Spann A, Borilovic J, de Witte L, Hawley M (2019). Understanding the care and support needs of older people: a scoping review and categorisation using the WHO international classification of functioning, disability and health framework (ICF). BMC Geriatr.

[17] Krause N, Liang J (1993). Stress, social support, and psychological distress among the Chinese elderly. J Gerontol.

[18] Tsutsui H, Ojima T, Ozaki N, Kusunoki M, Ishiguro T, Oshida Y (2015). Validation of the comprehensive international classification of functioning, disability and health (ICF) core set for Diabetes Mellitus in patients with diabetic Nephropathy. Clin Exp Nephrol.

[19] Horiuchi M, Takiguchi C, Kirihara Y, Horiuchi Y (2018). Impact of wearing graduated compression stockings on psychological and physiological responses during prolonged sitting. Int J Environ Res Public Health.

[20] Annor FB, Roblin DW, Okosun IS, Goodman M (2015). Work-related psychosocial stress and glycemic control among working adults with Diabetes Mellitus. Diabetes Metab Syndr Clin Res Rev.

[21] Moore RC, Eyler LT, Mausbach BT, Zlatar ZZ, Thompson WK, Peavy G (2015). Complex interplay between health and successful aging: role of perceived stress, resilience, and social support. Am J Geriatr Psychiatry.

[22] Hackett RA, Steptoe A (2017). Type 2 Diabetes Mellitus and psychological stress—a modifiable risk factor. Nat Rev Endocrinol.

[23] Van Eck M, Berkhof H, Nicolson N, Sulon J (1996). The effects of perceived stress, traits, mood states, and stressful daily events on salivary cortisol. Psychosom Med.

[24] Cohen S, Janicki-Deverts D, Miller GE (2007). Psychological stress and Disease. JAMA.

[25] Cohen S, Kamarck T, Mermelstein R. A global measure of perceived stress. J Health Soc Behav. 1983:385–96. 6668417

[26] Lazarus RS, Launier R (1978). Stress-related transactions between person and environment. Perspectives in interactional psychology.

[27] Ding Y, Yang Y, Yang X, Zhang T, Qiu X, He X (2015). The mediating role of coping style in the relationship between psychological capital and burnout among Chinese nurses. PLoS ONE.

[28] Chao YY, Zha P, Yang K, Dong X (2020). Association between physical function and perceived stress among US Chinese older adults. Am J Aging Sci Res.

[29] Zhang Z, Huang Q, Zhao D, Lian F, Li X, Qi W (2023). The impact of oxidative stress-induced mitochondrial dysfunction on diabetic microvascular Complications. Front Endocrinol.

[30] Tai LA, Tsai LY, Chiu YC (2023). Relation of environmental factors with activity limitations and participation restrictions in older adults with Diabetes Mellitus over time: an international classification of functioning framework perspective. BMC Geriatr.

[31] Shyu YIL, Yip PK (2001). Factor structure and explanatory variables of the Mini-mental State Examination (MMSE) for elderly persons in Taiwan. J Formos Med Assoc.

[32] Lawton MP, Brody EM (1969). Assessment of older people: self-maintaining and instrumental activities of daily living. Gerontologist.

[33] Lee HCB, Chiu HF, Kowk WY, Leung CM. Chinese elderly and the GDS short form: a preliminary study. Clin Gerontol J Aging Mental Health. 1993.

[34] Chan ACM (1996). Clinical validation of the geriatric depression scale (GDS) Chinese version. J Aging Health.

[35] Yesavage JA, Brink TL, Rose TL, Lum O, Huang V, Adey M, Leirer VO (1982). Development and validation of a geriatric depression screening scale: a preliminary report. J Psychiatr Res.

[36] Tong AY, Man DW (2002). The validation of the Hong Kong Chinese version of the Lawton Instrumental Activities of Daily Living Scale for institutionalized elderly persons. OTJR: Occup Particip Health.

[37] Hayes AF (2009). Beyond Baron and Kenny: statistical mediation analysis in the new millennium. Commun Monogr.

[38] Baron RM, Kenny DA (1986). The moderator–mediator variable distinction in social psychological research: conceptual, strategic, and statistical considerations. J Pers Soc Psychol.

[39] Preacher KJ, Hayes AF (2008). Asymptotic and resampling strategies for assessing and comparing indirect effects in multiple mediator models. Behav Res Methods.

[40] McEwen BS, Wingfield JC (2003). The concept of allostasis in biology and biomedicine. Horm Behav.

[41] Deschênes SS, Burns RJ, Schmitz N (2015). Associations between depression, chronic physical health conditions, and disability in a community sample: a focus on the persistence of depression. J Affect Disord.

[42] Wu CY, Terhorst L, Karp JF, Skidmore ER, Rodakowski J (2018). Trajectory of disability in older adults with newly diagnosed Diabetes: role of elevated depressive symptoms. Diabetes Care.

[43] Gonzalez JS, Peyrot M, McCarl LA, Collins EM, Serpa L, Mimiaga MJ (2008). Depression and Diabetes treatment nonadherence: a meta-analysis. Diabetes Care.

[44] Sinha R, Jastreboff AM (2013). Stress as a common risk factor for obesity and addiction. Biol Psychiatry.

[45] Meltzer H, Bebbington P, Brugha T, McManus S, Rai D, Dennis MS (2012). Physical ill health, disability, dependence and depression: results from the 2007 national survey of psychiatric morbidity among adults in England. Disabil Health J.

[46] Geneen LJ, Moore RA, Clarke C, Martin D, Colvin LA, Smith BH. Physical activity and exercise for chronic pain in adults: an overview of Cochrane Reviews. Cochrane Database Syst Rev. 2017;4. 10.1002/14651858.CD011279.pub3 PMC5461882 28436583

[47] Smith PJ, Merwin RM (2021). The role of exercise in management of mental health disorders: an integrative review. Annu Rev Med.

[48] Achterberg M, Dobbelaar S, Boer OD, Crone EA (2021). Perceived stress as mediator for longitudinal effects of the COVID-19 lockdown on wellbeing of parents and children. Sci Rep.

[49] Zhang S, Zou L, Chen LZ, Yao Y, Loprinzi PD, Siu PM (2019). The effect of Tai Chi Chuan on negative emotions in non-clinical populations: a meta-analysis and systematic review. Int J Environ Res Public Health.

[50] Rod NH, Kristensen T, Lange P, Prescott E, Diderichsen F (2012). Perceived stress and risk of adult-onset Asthma and other atopic disorders: a longitudinal cohort study. Allergy.

[51] Siu OL, Lo BCY, Ng TK, Wang H. Social support and student outcomes: the mediating roles of psychological capital, study engagement, and problem-focused coping. Curr Psychol. 2021:1–10. 10.1007/s12144-022-03339-w PMC9209831 35756898

[52] Lehrer HM, Janus KC, Gloria CT, Steinhardt MA (2017). Personal and environmental resources mediate the positivity-emotional dysfunction relationship. Am J Health Behav.

[53] Zhou J, Yang Y, Qiu X, Yang X, Pan H, Ban B (2016). Relationship between anxiety and burnout among Chinese physicians: a moderated mediation model. PLoS ONE.

[54] Abdullah MF, Mohd Nor N, Mohd Ali SZ, Ismail Bukhary NB, Amat A, Abdul Latif L (2011). Validation of the comprehensive ICF core sets for Diabetes Mellitus: a Malaysian perspective. Ann Acad Med Singap.

